# Evaluation of Chemical Composition of Two Linseed Varieties as Sources of Health-Beneficial Substances

**DOI:** 10.3390/molecules24203729

**Published:** 2019-10-16

**Authors:** Silvia Tavarini, Antonella Castagna, Giuseppe Conte, Lara Foschi, Chiara Sanmartin, Luca Incrocci, Annamaria Ranieri, Andrea Serra, Luciana G. Angelini

**Affiliations:** 1 Department of Agriculture, Food and Environment, University of Pisa, 56124 Pisa, Italy; antonella.castagna@unipi.it (A.C.); giuseppe.conte@unipi.it (G.C.); lara.foschi@unipi.it (L.F.); chiara.sanmartin@unipi.it (C.S.); luca.incrocci@unipi.it (L.I.); anna.maria.ranieri@unipi.it (A.R.); andrea.serra@unipi.it (A.S.); luciana.angelini@unipi.it (L.G.A.); 2 Interdepartmental Research Center “Nutraceuticals and Food for Health”, University of Pisa, 56124 Pisa, Italy

**Keywords:** flaxseed, oil, fatty acids, phenols, carotenoids, tocopherols, tocotrienols, seed yield

## Abstract

Linseed (*Linum usitatissimum* L.) is becoming more and more important in the health food market as a functional food, since its seeds and oil represent a rich source of bioactive compounds. Its chemical composition is strongly correlated with, and dependent on, genetic characteristics. The aim of this study was to evaluate the variation in seed yield, oil content, fatty acid composition and secondary metabolite profiles between a low-linolenic linseed variety, belonging to the Solin-type group (Solal), and a high-linolenic traditional one (Bethune), cultivated, both as spring crops, in open field conditions of Central Italy. The achieved results pointed out the different behavior of the two varieties in terms of growth cycle, oil content, and some important yield components, such as capsule number per plant and thousand seed weight. There were also significant differences in seed composition regarding total phenols, total flavonoids, antioxidant activities as well as in carotenoid, tocopherol, and tocotrienol profiles between the two varieties. In particular, Solal was characterized by the greatest contents of oil, phenols, flavonoids, α- and δ- tocotrienol, together with the highest antioxidant activity. Bethune, on the contrary, showed the highest amounts of carotenoids (lutein and β-carotene). These results indicate a clear effect of the genetic characteristics on the biosynthesis of these secondary metabolites and, consequently, on the related antioxidant activity. Our findings suggest that the mutation process, responsible for the selection of the low-linolenic cultivar, is able to modify the biosynthetic pathways of carotenoids and phenolics.

## 1. Introduction

In the last two decades, linseed (*Linum usitatissimum* L.), has been gaining more and more attention, since new perspectives for this crop are open as a source of high-added value raw material, for both food and industrial applications. Linseed usage for human consumption dates back to very ancient times during which it was used, not only for fiber cultivation or ‘manufactures’ production (drying oils, paints, coatings and printing inks), but also for medical purposes, as well as for nutritional products [1]. Nowadays, with the increased consciousness of consumers towards the strong relationship between food and health, linseed started to be considered a very promising functional food, due to the presence, in its seeds, of high amount of nutrients and biologically active compounds, such as fatty acids (specifically α-linolenic acid (ALA)), phytoestrogenic lignans (secoisolariciresinol diglycoside (SDG)), high quality proteins, dietary fibers and phenolic compounds [2,3,4]. Among these, ALA is one of the essential polyunsaturated fatty acids (PUFAs) representing the main functional component of linseed. It is clearly demonstrated that linseed represents the best ω-3 fatty acid source in vegetarian diets [5], having also the possibility to be used as additive in the preparation of many dietary products [6]. Even, if linseed chemical composition is strongly correlated with, and dependent on, genetic characteristics, growing conditions and crop management, generally the lipid content ranged between 37 and 45 g 100 g^−1^ of seed [3,7,8], and PUFAs (mainly α-linolenic and linoleic acid) represent more than 70% of total fatty acids [9]. Besides PUFAs, linseed is characterized by the presence of high amount of phenols, among which the most abundant are phenolic acids (mainly ferulic acid, chlorogenic acid, and gallic acid), flavonoids (belonging to the sub-group of flavones) and lignans [10,11]. All together, these components are responsible for important anticancer and anti-oxidative properties [4,5,6]. Finally, linseed oil contains carotenoids such as β-carotene [1] and water-soluble and fat-soluble vitamins, mostly represented by vitamin E, in its γ- tocopherol form, responsible for protection of cell proteins and fats against oxidation processes [3,12]. For all these important properties, linseed and linseed virgin oil are included in the 9th Edition of the European Pharmacopoeia. At the same time, linseed represents a valuable cosmetic raw material (i.e., flower extract, hull extract, seed extract, seed flour, seed oil, and many others), according to CosIng database. 

Although traditional linseed oil is naturally high in antioxidant compounds, it easily gets oxidized. This oil, in fact, has a high sensitivity to heat, light and oxygen exposure due to its great ALA content, responsible for rancidity and poor sensory quality, as a consequence of alteration of nutritional content [13,14]. Therefore, in order to obtain an enhancement in oxidative stability of linseed oil, genetically improved varieties have been developed with higher and more stable nutraceutical composition, modifying fatty acid profile and, thus, creating more suitable products for the food market. Considering this aspect, a linseed variety-group with reduced ALA content to a quantity less than 5% has been developed by using chemical mutagens, responsible for blocking the conversion process from double-unsaturated linoleic acid (ω-6 fatty acid) into triple-unsaturated linolenic acid [15]. The resulting new group of varieties, named Solin-type (trade name Linola), consists in low-linolenic mutants with greatly elevated levels of linoleic acid, ranging from 65% to 76% depending on variety and growing conditions, as well as higher levels of palmitic and oleic acid than traditional linseed varieties [16]. 

Therefore, in this study a low-linolenic variety, belonging to the Solin-type group (Solal), and a traditional linseed one rich in linolenic acid (Bethune) were compared, in order to assess their agronomical and qualitative characteristics. Through an open field experiment, carried out in 2015 growing season in Central Italy, plant growth, biometric characteristics, yield and yield components, together with seed characterization (oil and protein content, fatty acid composition and secondary metabolite profile), were investigated. 

## 2. Results and Discussion

### 2.1. Weather Conditions, Crop Growth and Yield

The coastal plain of Pisa province is characterized by Mediterranean climate, with minimum low temperatures in January (2 °C as mean monthly value), and maximum high temperatures in July (29 °C as mean monthly value). Rainfalls are mainly concentrated in autumn and spring time (941 mm year^−1^). During summer a dry period generally occurs from July to half August, with low rainfall and high air temperatures. 

The description of crop development, from emergence to seed maturity, has been followed in the two varieties. The timing and duration of each phenological phase, together with the corresponding growing degree days (GDD), accumulated rainfall and mean temperatures registered for each phenological stage, are reported in Table 1. Precipitation and temperature patterns were generally consistent with historical data. Cumulative amount of rainfall from planting to harvest (around 99 mm) was able to meet the water requirement of both linseed varieties. From seedling emergence to seed maturity, the minimum temperature increased from 7 to 15 °C and, similarly, maximum temperatures went from 20.5 to 31.5 °C. Both varieties were able to emerge 10 days after sowing (the day of emergence was set at 70% seedlings with unfolded cotyledons). The main differences in the cycle length were observed for the start of flowering, seed development and maturity, and, consequently, for the total growth cycle (Table 1). The evaluation of the entire growth cycle defined both the two varieties as fast-growing crops, when cultivated in spring sowing. In fact, the two varieties ended up their growing cycle just after 91 and 96 days from emergence to harvest, for Bethune and Solal respectively, with a thermal time equal to 1124 °C and 1224 °C. These findings revealed that Bethune was earlier than Solal, with shorter PGS duration, except for the time from end of flowering to seed development, probably due to the lower temperatures experienced during this stage. On the other hand, Solal during seed development experienced an early summer drought with mean maximum temperatures higher than that experienced by Bethune, with associated lower precipitations that, however, have not negatively influenced the performance of this variety. 

Regarding biometric characteristics (Table 2) evaluated at full maturity, no significant differences were detected for plant height, to indicate that this parameter was not affected by the genetic characteristics of the two studied varieties. Results obtained for plant height were in accordance to previous experiments carried out in Mediterranean environments, showing plant height values ranging from 0.4 to 0.5 m [17,18,19], with shorter plants in spring sowings (shorter growing cycle) as compared to autumn sowings (longer growing cycle) [20].

A significant lower plant density at harvest was registered for Solal than Bethune (Table 2). It is well known that linseed is characterized by great phenotypic plasticity, with a great ability to respond to changed spacing. Linseed, in fact, can compensate for low plant populations through extensive branching and increased size [21,22,23]. 

Several yield reports for linseed, grown in various environments, demonstrated that a low number of plants is balanced by increased capsules/plant due to branching [21,22,23,24,25]. Accordingly, in our experiment, seed yield was not significantly different between the two varieties, being capsules/plant and 1000 seed weight higher in Solal than Bethune. Bethune at higher population density, showed a shorter duration of green leaf area with less effective assimilatory capacity during capsule and seed growth. No differences in seed/capsule between the two cultivars have been observed.

It is also worth noticing that seed yields recorded for the two varieties in spring sowing in Central Italy were definitely lower than seed yields reported in other studies at higher latitudes of Poland and Finland [26,27], or in the same environmental conditions, but adopting an autumn sowing [19]. Our results were probably dependent on the shorter growing cycle, with higher temperatures and lower rainfall.

No differences were observed also for harvest index, revealing a mean value of 0.35 between the two varieties perfectly falling inside the range reported by D’Antuono and Rossini [28] based on the study of linseed cultivation in different Italian environments. When grown in spring sowing, linseed has been demonstrated to suffer heat stress during grain formation, determining poor production with minimum harvest index at higher temperature [29]. At the same time, a higher harvest index in fully irrigated crops was observed [30], as well as harvest index reduction at severe drought stress [31].

Mean seed oil content was significantly affected by the variety with the highest value recorded for Solal. It is reported that the linseed content ranges between 34 and 45% depending on geographical area, genotype and environmental conditions [32,33]. Despite the lower oil content recorded for Bethune, oil yield was not significantly different between the two varieties. Crude protein content was statistically higher for Bethune. As a general observation, the crude protein content was negatively correlated with oil content confirming previous findings [19,27].

### 2.2. Fatty Acid Composition

The fatty acid profile is reported in Table 3. Polyunsaturated FA represent the most abundant category with approximately 80% of total FA, while MUFA and SFA represent 13% and 6% respectively. Solal showed a significant higher level of SFA (6.99 vs. 5.72%) and a lower content (11.20 vs. 14.55%) of MUFA than Bethune. Regarding the single FA, α-linolenic acid was the most representative FA in the Bethune (64.02%), while linoleic acid was the most abundant FA in the Solal (77.82%). These results agree with characteristic already described in previous works [15,16]. The higher level of linoleic acid in Solal is the consequence of the use of chemical mutagens, responsible for blocking the conversion process from double-unsaturated linoleic acid (omega-6 fatty acid) into triple-unsaturated linolenic acid conversion [15]. Moreover, Solal showed a higher level of palmitic acid (C16:0) and a lower percentage of oleic acid (C18: 1c9). Despite the higher percentage of oleic acid in Bethune, the content (g × 100 g^−1^) is significantly higher in Solal as a consequence of the greater content of oil.

These results demonstrated that Bethune showed a better healthy property for the high level of omega-3, while the lower unsaturation level of Solal oil is optimal for storage. This conclusion is supported by the higher value of PUFA/SFA (22.68 vs. 11.69) and lower n6/n3 (0.13 vs. 19.61) ratios of Bethune than Solal.

### 2.3. Phenolic Compounds and Antioxidant Activity

**Figure 1:**
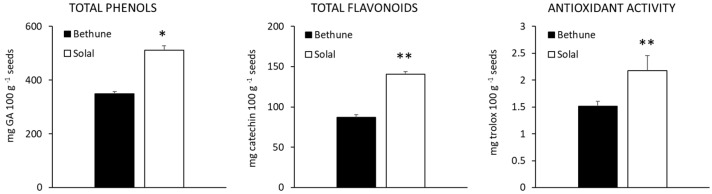
Concentration of total phenols (mg gallic acid equivalents 100 g^−1^ seeds) and total flavonoids (mg catechin equivalents 100 g^−1^ seeds), and antioxidant activity (mg trolox equivalents 100 g^−1^ seeds) of the two linseed varieties (Bethune and Solal). * = 0.05 ≤ *p*-value < 0.01; ** = 0.01 ≤ *p*-value < 0.001.

The concentration of total phenols and flavonoids, and the antioxidant activity of the hydroalcoholic extracts are reported in Figure 1. Both total phenols and flavonoids were more concentrated in Solal (511.60 and 140.38 mg 100 g^−1^ seeds, respectively) than in Bethune (349.70 and 87.25 mg 100 g^−1^ seeds, respectively). The flavonoid/phenol ratio was similar in the two varieties, ranging from 24.95 in Bethune to 27.44 in Solal. 

Accumulation of phenolic compounds may be affected by many factors, such as genetic background, pedoclimatic conditions and agronomic practices. Phenolic concentrations ranging from 61.76 to 85.24 μg GA/g were detected in six Indian linseed cultivars [34] while up to 474 mg GA/100 g were reported for Chinese cultivars [35]. These last values are consistent with the phenolic content detected in the present research. Similarly, Alu’datt et al. [36] found a total phenolic content variable from 0.90 to 4.69 mg/g, depending on the different extraction methods.

Phenolic compounds are recognized as important food metabolites, able to reduce the risk of the onset of many pathologies, as cardiovascular and neurodegenerative diseases and cancer [37,38]. These properties are attributed to anti-oxidative, anti-inflammatory, anti-mutagenic and anti-carcinogenic activities of phenolic compounds, coupled with their capacity to modulate key cellular enzyme function [37,38,39]. The antioxidant activity of the hydroalcoholic extracts of the two cultivars tested in the present study was in line with the results on phenol and flavonoid concentration. A higher activity was in fact displayed by Solal (+ 44% as compared to Bethune, Figure 1).

### 2.4. Carotenoids, Tocopherols and Tocotrienols 

**Figure 2:**
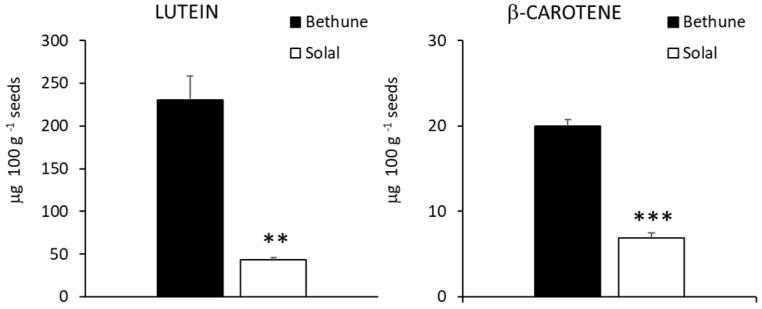
Concentration (μg 100 g^−1^ seeds) of lutein and β-carotene of the two linseed varieties (Bethune and Solal). ** = 0.01 ≤ *p*-value < 0.001; *** = *p*-value ≤ 0.001.

The carotenoid concentration is presented in Figure 2. 

Lutein is often reported to be the only carotenoid in linseed [40,41]. However, in the present study, both cultivars contained also small amounts of β-carotene, accounting for about 8% and 14% of total content in Bethune and Solal, respectively. Lutein concentration of Bethune was very similar to what reported by Franke et al. [40], while a 2.6-fold higher lutein level was measured in a US variety. Solal and Bethune presented significantly different concentration of carotenoids but, differently from what observed for phenolic compounds, Solal contained less carotenoids than Bethune (−81% of lutein and −65% of β-carotene), suggesting that the mutation process responsible for the selection of the low-linolenic cultivar affected in an opposite way the biosynthetic pathways of carotenoids and phenolics.

**Figure 3:**
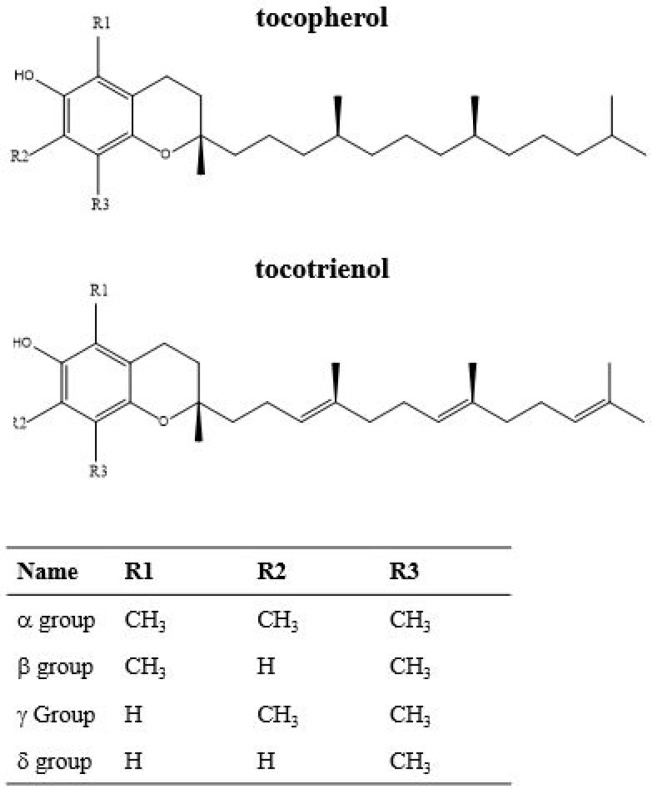
Tocopherol and tocotrienol chemical structures. The numbers and positions of methyl groups on the aromatic ring are reported in the table.

Tocopherols and tocotrienols can occur as four isomeric forms (α, β, γ and δ), differing in the number and position of methyl groups on the chromanol ring (Figure 3). 

**Figure 4:**
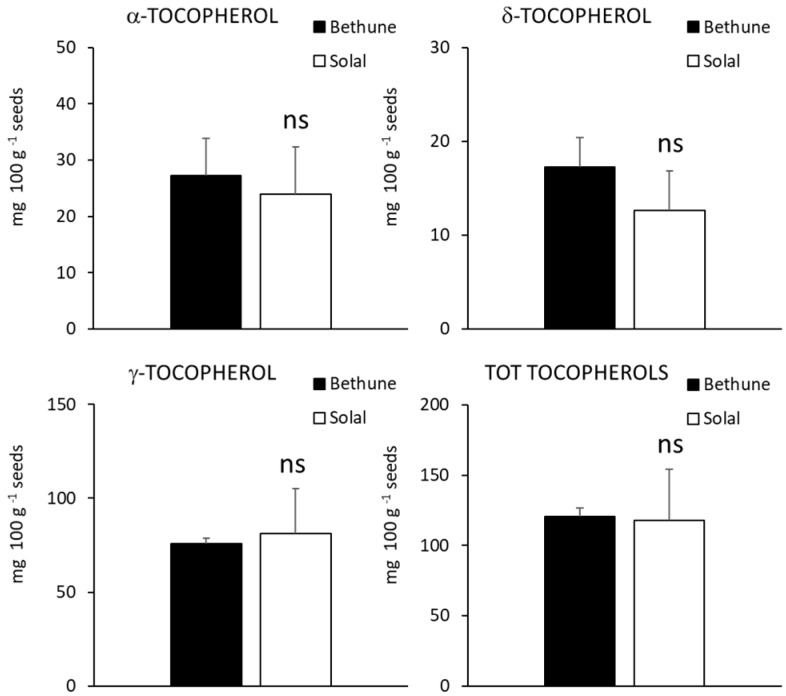
Concentration (mg 100 g^−1^ seeds) of α-, δ-, γ- and total tocopherols of the two linseed varieties (Bethune and Solal). n.s. = not significant.

**Figure 5:**
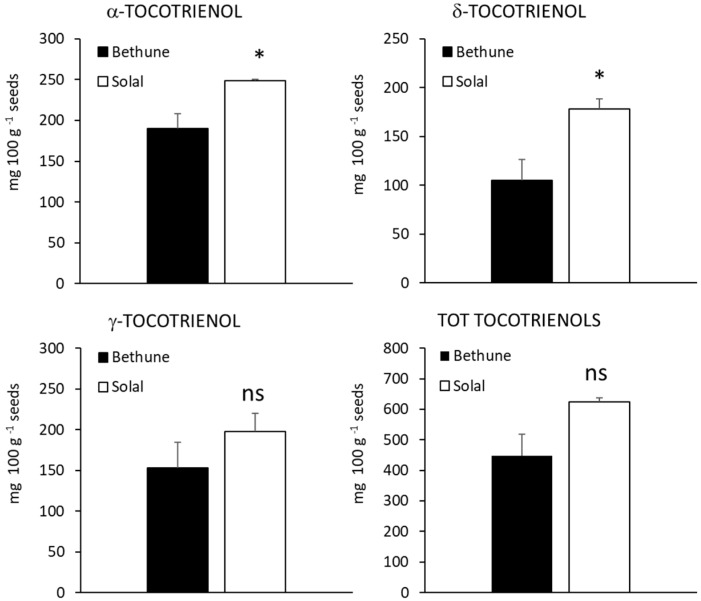
Concentration (mg100 g^−1^ seeds) of α-, δ-, γ- and total tocotrienols of the two linseed varieties (Bethune and Solal). n.s. = not significant; * = 0.05 ≤ *p*-value < 0.01.

Both linseed varieties contained α, γ and δ tocopherols and, in accordance with previous studies [42,43,44] the most abundant vitamer was the γ- form, that accounted for about 63% and 69% of total content in Bethune and Solal, respectively. Tocopherols are well known antioxidant compounds, able to prevent oxidation of unsaturated fatty acids present in the seeds and to provide protection against the damaging effects of free radicals in human cells. Moreover, tocopherols own anti-inflammatory and anti-cancer properties and are involved in prevention of low-density lipoprotein (LDL) oxidation and protection of blood vessels [45]. Though all vitamers are excellent antioxidants, α-tocopherol seems to be the form preferentially absorbed and accumulated in humans and the best chain breaker and peroxyl radical-scavenger [46], while γ-tocopherol is suggested to have a better capacity to detoxify nitric oxide reactive species [47]. No significant differences were detected in the levels of total tocopherols as well as in the amount of specific vitamers between the two linseed varieties (Figure 4). In both linseed varieties, α-tocotrienol was the most concentrated form (about 42% and 40% in Bethune and Solal, respectively; Figure 5), though the differences among the isomeric forms were less evident than for tocopherols. Alpha- and δ-vitamers were significantly more concentrated in Solal than in Bethune (+70% and +31%, respectively), similarly to what observed for phenolic compounds. Tocotrienols are receiving increasing attention due to their bioactivity and pharmacological potential, particularly as neuroprotectants and in the prevention of chronic disease [48,49]. Tocotrienols, thanks to their unsaturated side chain, penetrate, in fact, more efficiently into the tissues and better distribute in the lipid layers of the cell membrane, a character that makes them more powerful antioxidants than tocopherols [48,49]. 

## 3. Materials and Methods

### 3.1. Reagents and Standards

Acetonitrile HPLC grade (assay 99.9%) was purchased from Panreac Química S.A. (Barcelona, Spain); trifluoroacetic acid for HPLC (assay 99%) and formic acid for HPLC (assay 98%) were purchased from Sigma–Aldrich (Madrid, Spain). Folin–Ciocalteu reagent was purchased from Merck (Darmstadt, Germany). Water was purified by a Milli-Q water purification system from Millipore (Bedford, MA, USA).

The β-carotene and lutein standards used in this study was purchased from the Sigma Chemical Company (St. Louis, MO, USA). Other reagents including methanol, chloroform, n-hexane, petroleum ether (PE), diethyl ether (DE), acetone, ethanol, sodium chloride, potassium hydroxide, tri-ethyl amine and butylated hydroxyltoluene (BHT) were of analytical or HPLC grade.

### 3.2. Field Experiment and Plant Material

Two linseed varieties were compared, as spring crops, under field conditions, at the experimental Centre of the Department of Agriculture, Food and Environment of the University of Pisa, located at San Piero a Grado (Pisa province, Tuscany region, Central Italy, 3°40′N latitude; 10°19′E longitude, altitude 5 m a.s.l.), in 2015 growing season (from March to July). The area was characterized by flat land with an alluvial deep silt–loam soil, classified as Typic Xerofluvent soil, according to the USDA system [50]. The two linseed varieties, Bethune and Solal, were chosen on the basis of their fatty acid composition: Bethune, a brown-seeded linseed variety registered by the Canadian “Food Inspection Agency” in 1998, is characterized by a typical fatty acid profile with a high α-linolenic acid (ALA) content, while Solal, a yellow-seeded variety registered by the Italian Ministry of Agricultural, Food and Forestry Policies, belongs to the Solin-type (or Linola) linseed, showing an ALA content smaller than 5% (with a LA content of about 70%). Furthermore, these varieties differ also for important agronomic and morphological features. Bethune is generally characterized by a medium-late maturity, high rusticity, good tolerance to low temperatures, lodging and ‘pests’ resistance, with typically blue-light blue flowers and brown seeds. Furthermore, to date Bethune represents the only variety with a 93% of genome mapped [51,52,53,54]. Differently, Solal, even if characterized by a medium-late maturity cycle, low temperature and lodging resistance (similarly to Bethune), distinguishes for a yellow seed with a thinner tegument than the brown seeded varieties and light blue flowers. 

A field experiment was set up to compare the two varieties following a completely randomized block design with four replications. The plot size was 24 m^2^ (4.5 m × 6.0 m). Soil physical and chemical characteristics were assessed at the beginning of the experiment, collecting the soil samples at 30 cm depth in each plot. Soil pH determination was performed on a 1:2.5 soil:water suspension following McLean [55]. Electrical conductivity was measured by using a GLP-31 Crison conductimeter (52.93 electrode) (Montepaone s.r.l., San Mauro Torinese, Torino, Italy), at 20 °C. Total nitrogen was evaluated using the macro-Kjeldahl digestion procedure [56], available phosphorus by colorimetric analysis using the Olsen method [57] and cation exchange capacity was assessed according to Mehlich [58] method. Soil organic matter (SOM) was determined by multiplying the soil organic carbon (SOC) concentration by 1.724. SOC was measured using the modified Walkley–Black wet combustion method [59]. Finally, the Dreimanis [60] method was used for total CaCO_3_ evaluation, while the ammonium oxalate-titration method was used for active CaCO_3_ determination. The soil physical and chemical characteristics have been reported in Table 4. Changes in minimum, maximum and mean air temperatures and total rainfall were recorded throughout the field experiment by a weather station located nearby the experimental site.

Spring sowing of the two selected varieties was accomplished on the 19 March 2015. Wheat (*Triticum durum* Desf.) was the preceding crop. A deep soil ploughing (0.40 m) was carried out in the autumn of 2014, followed by disk harrowing for the seed bed preparation at the end of winter. A pre-planting fertilization was applied at rates of 80 ha^−1^ of both P_2_O_5_ (as triple perphosphate) and K_2_O (as potassium sulphate). The seed rate was 40 kg ha^−1^ with an intra-row spacing of 0.15 m for both varieties. Two top-dressed nitrogen applications were applied as ammonium nitrate at the rate of 40 kg N ha^−1^ each. 

### 3.3. Plant Sampling and Yield Evaluation

Phenological, biometric and productive characteristics were evaluated for each linseed variety. The phenology of the crops was periodically monitored, and the developmental stages analyzed were listed in Table 5, according to the specific description available for linseed proposed by Smith and Froment [61]. The length of each phenological growth stage (PGS) was calculated as the period of time occurring between one stage and the following one and expressed as duration days. The different PGS were also compared in terms of growing degree days (GDD), calculated by NOAA (National Oceanic and Atmospheric Administration) method. GDD were estimated following the equation GDD = _S1_Σ^S2^ (T_m_ − T_b_) where T_m_ was the mean daily temperature, T_b_ was the base temperature (5 °C was chosen as linseed base temperature according to McMaster et al. [62], and S1 and S2 as the starting phenological stage date and the selected following phenological stage date, respectively, expressed in Julian days. Cumulative rainfalls and minimum and maximum temperatures were also recorded for each PGS. Total growing cycle length (period of time occurring from the emergence to seed maturity), vegetative cycle (cycle length before flowering, from emergence to start of flowering) and reproductive cycle (cycle length after flowering, from start of flowering to seed maturity) were assessed. During plant growth, a preliminary sampling was carried out when the seeds of both varieties were plump and pliable (phenological phase number 8, sub-code 85 according to Smith and Froment [61]; see Table 5), in order to evaluate the dynamics of accumulation of bioactive compounds, within the seed. 

At full seed maturity, with a seed moisture lower than 9% for both varieties (seed moisture of 8.76% and 7.75%, for Bethune and Solal, respectively), final harvest was accomplished (6 July 2015). Biometric and productive parameters were evaluated on four randomized sample areas of 1 m^2^, manually harvested, within each plot for each linseed variety, excluding the outer rows. Plant density, plant height, total above-ground biomass, seed yield, capsules per plant and seeds per capsule were evaluated. Fresh weight was measured, and plants were subsequently allowed to dry in a ventilated oven (40 °C) for dry weight determination. Apparent harvest index (HI) was calculated as: (seed weight/mature plant weight) × 100. Thousand seed weight (TSW) was assessed according to ISTA method [63]. 

### 3.4. Crude Protein Content

Seed crude protein content was determined by means of the mini Kjeldahl method. Dried seed samples, after grounding, were analyzed for their nitrogen content by distillation and the crude protein content was determined multiplying the nitrogen content percentage for the conventional factor 6.25.

### 3.5. Fatty Acid Composition and Oil Content Determination

Fat content determination was achieved following the AOAC (Official Methods of Analysis) procedure for ether extract level, using ANKOM model XT10 extractor (ANKOM Technology, NY, USA).

Fatty acid composition was determined using the above described method. An acid trans-methylation was used to prepare fatty acids for the analysis following the procedure proposed by Christie [64] with some modification. Briefly, fatty acid methyl esters (FAME) were prepared by pouring 5 g of sample and 4.5 mL of 10% HCl methanolic solution into a 20 mL vial and mixed with a vibration mixer for 60 s. A nonadecanoic acid (1 mg) was added to the mix as internal standard. After 8 h, 5 mL of n-hexane were poured into the vial and the mixture was shaken for 1 min. The layers were allowed to separate, and the hexane fraction was injected to gas-chromatographic (GC) analysis. For the analysis was used a GC2010 Shimadzu gas chromatograph (Shimadzu, Columbia, MD, USA) equipped with a flame-ionization detector and a high polar fused-silica capillary column (Chrompack CP-Sil88 Varian, 152 Middelburg, the Netherlands; 100 m, 0.25 mm i.d.; film thickness 0.20 μm). Hydrogen was used as the carrier gas at a flow of 1 mL min^−1^. Split/splitless injector was used with a split ratio of 1:40. An aliquot of the sample was injected under the following GC conditions: the oven temperature started at 40 °C and held at that level for 1 min; it was then increased to 163 °C at a rate of 2 °C/min, and held at that level for 10 min, before being once again increased to 180 °C at 1.5 °C/min and held for 7 min, and then to 187 °C at a rate of 2 °C/min; finally the temperature was increased to 220 °C with a rate of 3 °C/min and held for 25 min. The injector temperature was set at 270 °C and the detector temperature was set at 300 °C. Individual FA methyl esters were identified by comparison with a standard mixture of 52 Component FAME Mix (Nu-Chek Prep Inc., Elysian, MN, USA).

### 3.6. Content of Total Phenols and Flavonoids 

Seed samples (0.5 g) were extracted with 5 mL of 80% methanol. The mixture was sonicated for 30 min, stirred for 30 min and centrifuged (30 min, 6000 rpm). The supernatant was recovered and stored at 4 °C, while the pellet was re-extracted twice with 5 mL of 80% methanol. After vacuum concentration by rotary evaporator, concentration of total phenols was determined using the Folin–Ciocalteu colorimetric method [65], recording the absorbance at 750 nm. Total phenol concentration was quantified using standard curve of gallic acid (0–250 mg L^−1^) and expressed as mg of gallic acid equivalents 100 g^−1^ seeds. 

Total flavonoids were quantified by aluminum chloride colorimetric method, following the procedure reported by Kim et al. [66]. Absorbance was read at 525 nm and results were expressed as mg of catechin equivalents 100 g^−1^ seeds, using a standard curve of catechin (0–250 mg L^−1^).

### 3.7. Determination of Antioxidant Activity 

Antioxidant activity was determined using ABTS (2,2′-azinobis-(3-ethylbenzothiazoline-6-sulfonic acid)) assay [67], based on the ability of antioxidants to reduce pre-formed radical cations of ABTS^+^ to ABTS. ABTS^+^ radicals were generated by 12–16 h incubation of 7 mM ABTS with 2.45 mM potassium persulfate in dark conditions. The solution was diluted to an absorbance of 0.700 ± 0.020 at 734 nm (30 °C), and 1 mL of ABTS^·+^ solution was mixed with adequately diluted sample aliquots to produce between 20–80% inhibition of the blank absorbance. The results were expressed as µmol Trolox equivalents 100 g^−1^ seeds, using a standard curve of Trolox (0–200 μmol L^−1^).

### 3.8. Quantification of Carotenoids, Tocopherols and Tocotrienols 

Fine-powdered seeds were incubated overnight with 5% KOH in methanol and 0.5% BHT under dark at room temperature in a rotary agitator and centrifuged at 5000× *g* (5 min at 4 °C). The supernatant was collected, mixed with one volume of water and two volumes of hexane and centrifuged to recover the upper organic phase. Extraction was repeated by adding hexane and the upper phase was pooled with the previous one. The solvent was evaporated by nitrogen flux and the dried sample was suspended in methanol/chloroform (1:1) solution and filtered through 0.22 µm PTFE syringe filters (Sartorius, Göttingen, Germany).

The analytical separation was performed by Spectra SYSTEM P4000 HPLC equipped with a UV 6000 LP photodiode array detector (Thermo Fisher Scientific, Waltham, MA, USA) and a Phenomenex Prodigy LC-18 ODS column (250 × 4.6 mm, 5 μm; guard column Phenomenex AJO-4287 C-18 ODS; Phenomenex srl, Castel Maggiore, Italy). Gradient elution was performed using solvent A (acetonitrile) and solvent B (hexane/methanol/dichloromethane, 1:1:1) as mobile phases at a flow rate of 0.8 mL/min. 

The gradient program for carotenoid analysis was: 0–10 min 0% to 44% B, 10–25 min, 44% to 61% B, 25–30 min, 61% to 0% B. The column was re-equilibrated in 0% solvent B for 5 min before the next injection. Carotenoids were detected at 450 nm, quantified using standard curves of lutein and β-carotene (0–250 mg L^−1^, Sigma Aldrich Chemical Co, St. Louis, MO) and expressed as µg 100 g^−1^ seeds.

The gradient program for tocopherols and tocotrienols was: 0–10 min 0% to 20% B, 10–25 min 20% to 61% B, 25–35 min 61% to 18% B, 35–36 min 18% to 0% B. The column was allowed to re-equilibrate in 0% solvent B for 5 min before the next injection. Compounds were detected at 280 nm, quantified using standard curves of commercial standards (0–250 mg L^−1^, Sigma–Aldrich Chemical Co, St. Louis, MO) and expressed as mg 100 g^−1^ seeds.

### 3.9. Statistical Analysis

For biometric and productive parameters, the *t*-test analysis was carried out, considering the genotype (G) as variability factor. GraphPad Prism 6 statistical package was used. 

Data derived from fatty acids composition were analyzed with the following linear model: y_ij_ = μ + L_i_ + ε_ij_ where: y_ij_ = dependent variables (fatty acids composition), L_i_ = fixed effect of the ith linseed species (B and S); ε_ij_ = random residual.

## 4. Conclusions

This study underlined the possibility to obtain, in the tested environment, an acceptable level of seed yields from linseed as spring crop, pointing out the different behavior of the two varieties in terms of cycle length and yield components. However, the differences between the two varieties were more pronounced in relation to qualitative and nutraceutical characteristics, indicating a clear effect of the genetic characteristics on the biosynthesis of the secondary metabolites here investigated, suggesting that the mutation process responsible for the selection of the low-linolenic cultivar can affect, in an opposite way, the biosynthetic pathways of carotenoids and phenolics. Even so, beyond the differences between the two varieties for the single bioactive compounds, our findings highlight the very high-quality standard oil. Besides PUFAs, differently represented in the two varieties due to the desired genetic diversity, the extensive biochemical analysis carried out in this study, resulted in the quantification and identification of important components with potential application in improvement of human health. These properties make the oil and seeds suitable candidates for the development of new branded healthy and functional foods, and appealing ingredients for cosmetic and pharmaceutical industry.

## Figures and Tables

**Table 1:** Main phenological growth stages (PGS) of the two linseed varieties (Bethune and Solal) expressed as duration days, growing degree days (GDD) and precipitation (rainfall) accumulated for each phenological period. Values are expressed as means of three observations. Minimum and maximum temperature resulted from mean values referred to each phenological period.

PGS	Duration		GDD (°C)		Cumulate Rainfall (mm)		T Min (°C)		T Max (°C)	
	Bethune	Solal	Bethune	Solal	Bethune	Solal	Bethune	Solal	Bethune	Solal
Emergence—start of flowering	42	49	385.30	478.90	53.20	66.40	7.42	7.92	20.50	21.23
Start-End of flowering	8	11	109.90	126.55	13.40	10.60	11.23	10.29	26.25	22.72
End of flowering—seed development *	33	27	494.95	451.65	19.20	21.20	13.32	14.69	26.68	28.76
Seed development *—seed maturity	8	9	133.80	166.65	12.80	1.00	13.69	15.52	29.76	31.51
Total growing cycle	91	96	1123.95	1223.75	98.60	99.20	10.41	10.78	24.02	24.45
Vegetative cycle (BF **)	42	49	385.30	478.90	53.20	66.40	7.42	7.92	20.50	21.23
Reproductive cycle (AF ***)	49	47	738.65	744.85	45.40	32.80	13.04	13.82	27.11	27.87

* seed plump and pliable; ** before flowering; *** after flowering.

**Table 2:** Yield and yield components (results are the means ± standard deviation of four replicates) in two linseed varieties (Bethune and Solal) at harvest (6 July 2015).

	Bethune	Solal	*p*-Value ^1^
Plant density (n. plant m^−2^)	240.00 ± 32.66	140.63 ± 10.18	**
Plant height (m)	0.45 ± 0.04	0.50 ± 0.03	n.s.
Total above-ground biomass (Mg ha^−1^)	1.90 ± 0.28	2.33 ± 0.36	n.s.
Seed yield (Mg ha^−1^)	0.80 ± 0.09	0.73 ± 0.04	n.s.
Capsules per plant (n.)	8.50 ± 0.04	14.13 ± 0.53	***
Seeds per capsule (n.)	7.91 ± 1.20	6.34 ± 1.63	n.s.
Thousand seed weight (g)	4.81 ± 0.02	5.69 ± 0.07	***
Harvest Index (HI)	0.38 ± 0.06	0.31 ± 0.01	n.s.
Oil content (% dry matter)	34.23 ± 0.89	41.33 ± 0.86	***
Crude protein content (%)	20.84 ± 0.22	20.49 ± 0.09	*
Oil yield (kg ha^−1^)	272.73 ± 50.99	302.19 ± 41.33	n.s.

^1^ n.s. = not significant; * = 0.05 ≤ *p*-value < 0.01; ** = 0.01 ≤ *p*-value < 0.001; *** = *p*-value ≤ 0.001.

**Table 3:** Major fatty acid composition (expressed as g 100 g^−1^ of seed and as g 100 g^−1^ of total FA) of the two linseed varieties (Bethune and Solal).

	Fatty Acids (g × 100 g^−1^ of Seed)				Fatty Acids (g × 100 g^−1^ of Total FA)			
	Bethune	Solal	SE ^1^	*p*-Value ^2^	Bethune	Solal	SE ^1^	*p*-Value ^2^
C16:0	0.84	2.00	0.06	***	4.20	5.50	0.24	***
C16:1c9	0.02	0.04	0.01	*	0.09	0.12	0.02	n.s.
C17:0	0.01	0.05	0.01	*	0.07	0.13	0.03	n.s.
C18:0	0.28	0.46	0.07	n.s.	1.40	1.27	0.15	n.s.
C18:1c9	2.77	3.78	0.18	***	13.91	10.42	1.50	*
C18:1c11	0.11	0.24	0.03	*	0.55	0.66	0.05	n.s.
C18:2c9c12	3.06	28.24	2.10	***	15.39	77.82	1.87	***
C18:3c9c12c15	22.74	1.44	0.89	***	64.02	3.96	1.64	***
SFA ^3^	1.14	2.54	0.07	***	5.72	6.99	0.24	***
MUFA ^4^	2.91	4.08	0.13	***	14.55	11.20	1.75	*
PUFA ^5^	25.86	29.68	0.95	***	79.40	81.78	1.63	n.s.
PUFA/SFA	22.68	11.69	0.84	***	13.88	11.69	1.63	n.s.
n6/n3	0.13	19.61	2.34	***	0.24	19.65	2.44	***

^1^ SE = standard error; ^2^ n.s. = not significant; * = 0.05 ≤ *p*-value < 0.01; ** = 0.01 ≤ *p*-value < 0.001; *** = *p*-value ≤ 0.001; ^3^ SFA = saturated fatty acids; ^4^ MUFA = monounsaturated fatty acids; ^5^ PUFA = polyunsaturated fatty acids.

**Table 4:** Physical and chemical characteristics (0–30 cm depth) of the soil at the beginning of the field experiment.

Clay (%)	18.4
Silt (%)	39.1
Sand (%)	42.5
pH (1:25)	8.15
Electrical conductivity (μS cm^−1^)	71.2
Total N (‰)	1.18
Organic matter (%)	1.8
Assimilable P (mg kg^−1^)	3.6
Cation exchange capacity (mEq 100 g^−1^)	11.9
Total CaCO_3_ (%)	33.3
Active CaCO_3_ (%)	2.8

**Table 5:** Growth stage key for linseed *Linum usitatissimum* L in spring sowing (2015 growing season).

Code ^a^	Definition	Date	
0: Germination and emergence (sub-code 05: emergence)	Emergence ^b^	29 March 2015	
5: Inflorescence emergence (sub-code 59: first flower formed)	Beginning of flowering	Bethune 10 May 2015	Solal 17 May 2015
6: Flowering and capsule formation (sub-code 69: end of flowering, all pedicles bearing capsules)	End of flowering	Bethune 18 May 2015	Solal 28 May 2015
8: Capsule and seed ripening (sub-code 85: capsules all yellow brown, but soft)	Seed development (seed plump and pliable)	Bethune 20 June 2015	Solal 24 June 2015
8: Capsule and seed ripening (sub-code 89: capsules brown, seed rattle in the capsule)	Seed maturity	Bethune 28 June 2015	Solal 3 July 2015

^a^ According to Smith and Froment [61]. ^b^ Sowing 19 March 2015; Sampling at harvest 6 July 2015.
